# A Framework to Guide Implementation of AI in Health Care: Protocol for a Cocreation Research Project

**DOI:** 10.2196/50216

**Published:** 2023-11-08

**Authors:** Per Nilsen, Petra Svedberg, Margit Neher, Monika Nair, Ingrid Larsson, Lena Petersson, Jens Nygren

**Affiliations:** 1 School of Health and Welfare Halmstad University Halmstad Sweden; 2 Department of Health, Medicine and Caring Sciences Linköping University Linköping Sweden

**Keywords:** artificial intelligence, AI, health care, implementation, process models, frameworks, framework, process model

## Abstract

**Background:**

Artificial intelligence (AI) has the potential in health care to transform patient care and administrative processes, yet health care has been slow to adopt AI due to many types of barriers. Implementation science has shown the importance of structured implementation processes to overcome implementation barriers. However, there is a lack of knowledge and tools to guide such processes when implementing AI-based applications in health care.

**Objective:**

The aim of this protocol is to describe the development, testing, and evaluation of a framework, “Artificial Intelligence-Quality Implementation Framework” (AI-QIF), intended to guide decisions and activities related to the implementation of various AI-based applications in health care.

**Methods:**

The paper outlines the development of an AI implementation framework for broad use in health care based on the Quality Implementation Framework (QIF). QIF is a process model developed in implementation science. The model guides the user to consider implementation-related issues in a step-by-step design and plan and perform activities that support implementation. This framework was chosen for its adaptability, usability, broad scope, and detailed guidance concerning important activities and considerations for successful implementation. The development will proceed in 5 phases with primarily qualitative methods being used. The process starts with phase I, in which an AI-adapted version of QIF is created (AI-QIF). Phase II will produce a digital mockup of the AI-QIF. Phase III will involve the development of a prototype of the AI-QIF with an intuitive user interface. Phase IV is dedicated to usability testing of the prototype in health care environments. Phase V will focus on evaluating the usability and effectiveness of the AI-QIF. Cocreation is a guiding principle for the project and is an important aspect in 4 of the 5 development phases. The cocreation process will enable the use of both on research-based and practice-based knowledge.

**Results:**

The project is being conducted within the frame of a larger research program, with the overall objective of developing theoretically and empirically informed frameworks to support AI implementation in routine health care. The program was launched in 2021 and has carried out numerous research activities. The development of AI-QIF as a tool to guide the implementation of AI-based applications in health care will draw on knowledge and experience acquired from these activities. The framework is being developed over 2 years, from January 2023 to December 2024. It is under continuous development and refinement.

**Conclusions:**

The development of the AI implementation framework, AI-QIF, described in this study protocol aims to facilitate the implementation of AI-based applications in health care based on the premise that implementation processes benefit from being well-prepared and structured. The framework will be coproduced to enhance its relevance, validity, usefulness, and potential value for application in practice.

**International Registered Report Identifier (IRRID):**

DERR1-10.2196/50216

## Introduction

Artificial intelligence (AI) has the potential to revolutionize health care delivery [1]. AI refers to the simulation or approximation of human intelligence in machines, such as perception, reasoning, and decision-making [2]. It can be used for a variety of applications in health care, for example, diagnosis, treatment planning, disease prediction, and personalized medicine [3-5]. AI can analyze large amounts of data quickly and accurately. This can be especially useful in health care for screening and diagnosis; AI can identify patterns and anomalies in medical images or laboratory results that human clinicians might miss or overlook [6]. AI can also be used to predict disease progression and recommend treatment options based on a patient’s individual characteristics and medical history [7]. AI can also be useful in the development of digital tools and interventions in health care [8]. AI-powered mobile apps and wearables such as smartwatches can provide patients with personalized health recommendations, monitor vital signs, and alert health care providers to potential problems. This can help to reduce the burden on health care systems and improve patient outcomes by enabling more proactive and personalized care.

Expectations for the use of AI-based applications to improve health care delivery and efficiency are high. However, this sector has been slow to make use of AI compared with other sectors of society, such as banking, transport, and retail [9-12]. The implementation of AI in health care has encountered various technological, individual, social, and organizational barriers. These barriers have been classified in different ways, for example, data related, methodological, technological, regulatory or policy related, human factor related, environmental, and organizational [13-15]. Several studies have concluded that the implementation of AI-based applications tends to be unstructured [16,17]. Research on strategies to overcome these barriers or how to best implement AI-based applications in health care is scant [17].

Research in implementation science on the introduction and use of technological innovations and other new practices has shown that implementation is often suboptimal [18]. Implementation tends to be a complex and challenging process because it requires changes in existing behaviors, practices, and routines, many of which are well established or ingrained [19]. There may also be competing priorities or resource constraints that make it difficult to prioritize the implementation of new practices. To address these challenges, implementation science emphasizes the importance of planning for implementation from the outset to create conditions conducive to implementation and carrying out implementation processes in a structured, well-planned, and orderly way [20]. Process models are a key tool in implementation science, providing guidance for well-prepared and structured implementation by describing the key considerations and activities that are important to take into account during the implementation process [21]. These models help to ensure that implementation proceeds in a way that is systematic and focused on achieving the desired outcomes.

Interest in using AI in health care has increased in recent years, but there is a lack of knowledge to guide the process of implementing various AI-based applications in this setting. Therefore, the aim of this protocol is to describe the development, testing, and evaluation of a framework (“Artificial Intelligence-Quality Implementation Framework”; AI-QIF) intended to guide decisions and activities related to the implementation of various AI-based applications in health care. The target group of the framework are those who make decisions about, lead, or are involved in change processes in connection with the implementation of AI-based applications in health care.

## Methods

### Overall Design

This project will use a cocreation design [22] to develop, test, and evaluate a framework to guide the implementation of AI-based applications in health care. This will be carried out in 5 phases by Halmstad University researchers in collaboration with various stakeholders (Table 1). Cocreation involves collaboration between researchers and stakeholders for a creative problem-solving process, from design, production, and implementation to evaluation of solutions [22]. Cocreation has been found to improve the quality, usability, and social acceptance of new solutions [23]. In this project, participants with different roles in relation to AI implementation will offer diverse insights by means of interviews and informal conversations. The aim of the cocreation approach is to obtain a more holistic view of the form, content, and potential value of the framework that will be developed and to ascertain its usability in health care practice.

**Table 1:** The 5 phases of the project to develop, test, and evaluate the AI-QIF^a^.

Phase and time period	Aim of the phase	End product of the phase	Data collection	Data analysis	Cocreation aspects
I: January to June 2023	To produce an AI^b^-adapted version of QIF^c^	A preliminary version of a Swedish-language AI-QIF (AI-QIF 1.0)	Inspection of the Swedish QIF version developed by the Public Health Agency of Sweden; consultations with QIF experts at the Public Health Agency of Sweden.	No formal analysis. Discussions in the research team regarding the adaptation of QIF for AI purposes.	QIF experts at the Public Health Agency of Sweden
II: June to December 2023	To enrich a preliminary version of AI-QIF and to make sure it is grounded in empirical AI research	An initial version of a Swedish-language AI-QIF (AI-QIF 1.2) presented as a mockup of a web-based application	Secondary data in the form of published studies concerning AI implementation and transcripts of 120 interviews conducted by Halmstad University researchers.	Deductive mapping of information to the 14 steps of QIF.	Managers, practitioners, and developers from health care as well as individuals from academia and the business sector with AI knowledge
III: September to December 2023	To incorporate organization- and management-relevant AI aspects	A prototype AI-QIF with an intuitive user interface (AI-QIF 1.3)	Individual interviews with managers in Region Halland involved in AI-relevant decision-making, national AI experts, and patients. Estimated interviews, n=20.	Qualitative content analysis.	Managers in Region Halland involved in AI-relevant decision-making and national AI experts
IV: January to June 2024	To investigate the usability of an AI-QIF prototype under real-world conditions	A proposed final version of the framework (AI-QIF 2.0)	Individual interviews with managers and AI-users in Region Halland involved in 3 AI cases. Estimated interviews, n=20.	Qualitative content analysis.	Managers and AI users involved in 3 AI cases in Region Halland
V: August to December 2024	To evaluate the real-world use of the final AI-QIF and investigate barriers and facilitators to using the AI-QIF when implementing various AI applications	A description of the development of the AI-QIF and guidelines for its use in practice based on the key barriers and facilitators to its use in routine practice that have been identified	Individual interviews with approximately 20 users of the AI-QIF in 5 regions in Sweden.	Qualitative content analysis, inductive and deductive, the latter based on Capability-Opportunity-Motivation-Behaviour.	Users of AI-QIF in 5 regions

^a^AI-QIF: Artificial Intelligence-Quality Implementation Framework.

^b^AI: artificial intelligence.

^c^QIF: Quality Implementation Framework.

### Quality Implementation Framework as the Basis for the Initial Development of the Framework

The AI implementation framework will be based on the Quality Implementation Framework (QIF), a process model in implementation science aimed at guiding implementation processes. The QIF describes a number of activities and considerations that should be undertaken when implementing new practices. It was developed by Meyers et al [24] to achieve an improved understanding of “the nature of the implementation process.” Meyers et al [24] posited that “quality implementation” is “putting an innovation into practice in such a way that it meets the necessary standards to achieve the innovation’s desired outcomes.” In total, 25 theories, models, and frameworks across multiple research and practice areas were examined. A synthesis of the frameworks yielded 14 activities (referred to as steps) to be carried out in the implementation process. These steps are clustered into a 4-phase temporal sequence [25].

The first phase of the QIF is an initial consideration regarding the host setting, with work focusing primarily on making sure there is a fit between the innovation and the host setting. The second phase involves creating a structure for implementation and emphasizing the importance of creating an organized structure to oversee the implementation process. This can involve, for example, having a clear plan for implementing the innovation and identifying a team of qualified individuals who will take responsibility for the process. The third phase is aimed at creating an ongoing structure once implementation begins, for example, providing required technical assistance, coaching and supervision, and creating supportive feedback mechanisms for the users of the innovation. The fourth phase is aimed at learning from the implementation of the innovation to improve future applications [25].

The QIF has many strengths that were important for choosing this process model as a starting point for the AI-QIF. It has been widely used and shown to be feasible in many different types of settings, for example, studies on the implementation of a primary health care system improvement initiative in South Africa [26], electronic medical records for clinical and research purposes in Malaysia [27], an oral screening tool for pediatric cardiologists in the United States [28], and a mindfulness-informed social and emotional learning program in elementary schools in England [29]. The flexible application of the QIF underscores the model’s adaptability and usability. Furthermore, the QIF has a broad scope and is not restricted to any specific “implementation object.” Rather, the “object” can be any practice (innovation, intervention, program, routine, service, etc) that is introduced into an organization. Other process models in implementation science have a considerably narrower scope, for example, focusing on how research findings are used by nurses in their everyday practice [21].

The QIF is comprehensive, yet it provides fairly detailed guidance concerning important activities and considerations for successful implementation. This is in contrast to many other process models in implementation science that provide considerably more generic guidance, for example, describing an overarching process of how research is transformed into action [21]. A further strength of the QIF is that it has been translated into Swedish and used in Swedish health care by the Public Health Agency of Sweden [30]. The agency is an expert state authority under the Government’s Ministry of Social Affairs with national responsibility for public health issues. The agency aims to protect the population against communicable diseases and other health threats, developing general guidelines and recommendations [31]. Agency staff tasked with developing an updated Swedish version of the QIF have recently collected feedback from QIF users in various settings, which they have used for some modifications to the Swedish version.

### Phase I: Repurposing the QIF for Use in an AI Implementation Context

The first phase is aimed at verifying the validity of the QIF process model as the starting point for producing the first version of an AI-adapted version of the Swedish-language AI implementation process model (AI-QIF 1.0). This work is similar to the psychometric investigation of content validity and face validity that is done for various assessment instruments [32]. This preliminary version will be collated from the original QIF (in English) and current Swedish versions of the QIF.

Data will be collected in 2 ways. First, we will examine the Swedish QIF translation developed by the Public Health Agency of Sweden, comparing and contrasting the original QIF with the Swedish version item by item. Potential differences will be discussed with representatives of the team at the Public Health Agency of Sweden, who developed the Swedish version.

Second, we will draw on feedback documented by the Public Health Agency of Sweden from testing of the QIF by Region Dalarna and 15 municipalities in Dalarna. The framework is also used by other regions, but the Public Health Agency has not yet received feedback from them. Comments and suggestions for revisions have been collected by the team (n=3) at the agency who are in charge of the QIF. We will consult with QIF-knowledgeable experts in this team concerning lessons learned from this hands-on use of the QIF, for example, in terms of potential difficulties in interpreting items or whether important aspects were perceived to be missing from the Swedish QIF model.

### Phase II: Developing a Mockup of the AI-QIF

The aim of the second phase is to enrich the preliminary version of the Swedish-language AI-QIF and to make sure it is grounded in empirical AI implementation research. The work is largely similar to face and content validity evaluation that is part of instrument development [32]. This is achieved by accounting for and integrating empirical findings from two sources: (1) AI implementation cases described in studies identified through literature reviews [17,33] and (2) interview studies involving various stakeholders involved in or affected by the implementation of AI applications in health care [34]. The end product of phase II will be an adapted version of a Swedish-language AI-QIF (AI-QIF 1.1) presented as a mockup of a web-based application.

First, we will examine AI literature of relevance for the AI-QIF by identifying AI implementation cases in studies identified in literature reviews of AI implementation studies [17,33] and examining the studies in question. This will enable us to identify overlap between the AI-QIF and existing knowledge about barriers and strategies concerning the implementation of AI in health care as well as possible differences and other aspects to take into account when implementing AI in health care.

Second, we will draw on a secondary analysis of 120 interviews conducted with stakeholders such as managers, practitioners, and developers from health care as well as academia and business sector individuals with AI knowledge [34]. The interviews will focus on, among other AI applications, mortality prediction in the emergency department, prevention of mental health problems among young adults, digital triage in primary care, and reducing the risk of readmission of heart failure patients. The purpose of analyzing these interviews is to identify AI-specific implementation barriers and strategies that might be relevant to integrate into the framework. The interviews address experiences and perceptions held by stakeholders concerning the implementation of AI in general and various specific AI-based applications.

Findings from the 2 sources (ie, AI implementation cases in the literature and interviews) will be combined and analyzed in a deductive mapping process [35] to the contents of the 14 steps of the first version of the AI-QIF (from phase I). In the first step of the analysis, an inductive approach will be undertaken to identify barriers and strategies for the implementation of AI in practice. In the second step, a deductive approach will be undertaken to map these barriers and strategies to the 14 steps in the AI-QIF. If deemed necessary, parts of AI-QIF can be adjusted to fit the needs of stakeholders by adjusting, adding, or removing steps or changing the wording in the framework to fit AI implementation. All authors will be involved in the entire analysis process, thus enabling a variety of interpretations of the material before consensus is reached among all authors.

Third, the results of phase II will be used to further adapt the Swedish-language AI-QIF (AI-QIF 1.2) and will be presented as a mockup of a web-based application.

### Phase III: Developing a Prototype of the AI-QIF

The aim of phase III is to incorporate organization- and management-relevant AI aspects based on input from national AI experts and health care managers in Region Halland who are involved in AI-relevant decision-making and deployment of AI-based applications in health care in this region (regions are responsible for the provision of health care in Sweden). The goal is also to achieve a comprehensive face-validity check [32] of an intuitive and user-friendly prototype of the AI-QIF (AI-QIF 1.3).

We will develop an interview guide and conduct interviews with AI experts and managers on usability and feasibility issues around the potential use of the AI-QIF in practice. Purposive sampling will be used to identify and select individuals who can provide rich information to achieve the objective of developing the AI-QIF prototype. Approximately 20 interviews are tentatively planned, but the actual number of interviews will be guided by the information power principle [36], meaning that the larger information the sample holds relevant for the study, the lower amount of interviews is needed, and vice versa. The interviewees’ experiences and perceptions will improve the fit of the framework within the context in which it will be used. The interviews will potentially function as a needs assessment, thus adding to our understanding of which organizational actors could be involved in the implementation of AI.

The interviews will also cover which terminology is experienced as appropriate, clear, and concise enough for users in these organizational capacities. The interviews will also invite participants to highlight their preferred order of the steps, which AI-QIF steps are perceived as more or less important, and which steps might be missing or redundant.

The interviews will be analyzed using qualitative content analysis [35], and the information from the interviews will be applied to the development of the prototype, which will be tested and verified iteratively with the informants to ensure that consensus is reached on how the steps should be understood and acted upon.

### Phase IV: Usability Testing of the AI-QIF Prototype

Phase IV involves usability testing of the prototype of the AI-QIF developed in phase III [37]. The aim is to investigate experiences from and functionality of its use under real-world conditions. Usability testing at the prototype stage presents opportunities to check the user experiences and functionality before a final version is developed. The end product of phase IV will be the proposed final version of the framework (AI-QIF 2.0) for investigation in phase V.

For this purpose, 3 real-world AI implementation cases will be selected in close collaboration with relevant managers in Region Halland to ascertain that appropriate cases are chosen to enable timely testing of the framework before implementation starts. The following cases are planned: (1) optimized work practices and patient flows for improved care of patients with heart failure based on the implementation of an AI-based application that enables prediction of readmission risk; (2) new tools for documentation, follow-up and optimization of care flows, and management of patients with long-term wounds based on an AI-based application for analysis of and guidance for optimization of individualized wound care; and (3) introduction of interventions and resources for increased accessibility to primary care and specialist mental health care for young individuals with mental health problems based on AI-based applications for health monitoring, identification of risk groups, and mediation of care.

Key informants in each AI case will be recruited for structured interviews focusing on usability issues [38], with both Region Halland managers and users involved in any of the 3 selected cases. The aim is to investigate their experiences of using the prototype framework when implementing AI-based applications in health care. Approximately 20 interviews (managers and users) are tentatively planned, but sampling will be based on the information power principle [36]. The interviews will be analyzed using qualitative content analysis [35]. Information from these interviews will provide input for potential modifications of the framework and the prototype for the digital web application.

### Phase V: Evaluating the Use of the AI-QIF

The aim of the final phase is to evaluate the real-world use of the final version of the AI-QIF and to investigate barriers and facilitators to using the AI-QIF when implementing various AI applications. The users will be asked to reflect on how they used the AI-QIF. The end product of phase V will be a description of the development of the framework and guidelines for its use in practice based on the key barriers and facilitators to its use in routine practice that have been identified.

A qualitative explorative design will be used based on individual semistructured interviews with a sample of approximately 20 framework users in 5 regions. The regions will be purposively selected with regard to geographical location (regions in different parts of Sweden) and size in terms of populations (small and large regions). A combination of conventional (inductive) and directed (deductive) approaches to qualitative content analysis will be used [35]. The deductive analysis will use 3 predefined categories of the widely used implementation determinant framework, COM-B (Capability-Opportunity-Motivation-Behaviour). COM-B posits that behavior (B), for example, using the AI-QIF, is influenced by the person’s capability (C), opportunity (O), and motivation (M) to perform that behavior [38].

### Ethical Considerations

The study conforms to the principles outlined in the Declaration of Helsinki [39] and will fulfill the following requirements for research: information, consent, confidentiality, and safety of the participants, guided by the ethical principles of autonomy, beneficence, nonmaleficence, and justice. Ethical approval by the Swedish Ethical Review Authority (2020-06246, 2023-02581-02) is available for required parts in phases I and II. Ethical approval will be applied for the work to be conducted in phases III, IV, and V. All participants will receive written and oral information about the studies in which they are directly or indirectly involved. Participants will also be given information about the voluntary nature of the studies, confidentiality, and the ability to withdraw their consent at any time without having to justify why. All personal data will be registered according to the General Data Protection Regulation (GDPR2016/679), and the data will be stored in accordance with the Archive Act in Sweden (SFS1990:782). The results will be communicated to the participants and partners, and key findings will be fed back to participants and study sites to enable refinement of the developed framework. The results will be disseminated via publications in peer-reviewed journals and presentations at national and international conferences.

## Results

This project will be conducted within the frame of a research program “Toward Successful Implementation of Artificial Intelligence in Health Care Practice” [34] with the overall objective of developing theoretically and empirically informed frameworks to support AI implementation in routine health care. The program was launched in 2021 and has carried out numerous research activities, including scoping reviews, mapping of different stakeholder perspectives, and initiation of several case studies. Thus, the development of AI-QIF as a tool to guide the implementation of AI-based applications in health care will draw on knowledge and experience acquired from these activities.

The development process of the AI-QIF started in January 2023 (phase I) through collaboration with the Public Health Agency of Sweden, and the plan is to finish the tool in December 2024. The AI-QIF is under continuous development and refinement. Reporting will be done through scientific papers and various formats for more practical application in health care and in national efforts on promoting and supporting AI implementation in health care.

The insights gained during the development and refinement of the framework will be used as the foundation for parallel investments in regional capacity to increase the practical resources, competencies, partnerships, and organizational structures required to facilitate the implementation of AI-based applications. This work will be carried out in collaboration with representatives from academia, strategic partners from the business sector, and political and operational leaders and teams from the regional and municipal health care systems. The framework will be an integral part of a strategy toward the use of AI in health care (described as a “roadmap”), which is planned with strategic investments to support AI implementation and quality improvement in clinical health care practice [34].

## Discussion

There is little doubt that AI will play an important role in the future of health care, yet research has documented that the use of AI in health care faces many challenges in the “last mile” of the AI research and development process, that is, the introduction and routine use of AI-based applications in health care settings [40,41]. Numerous barriers to the implementation of AI in health care have been identified [9-14,17]. The AI-QIF described in this study protocol aims to facilitate the implementation of AI-based applications in health care based on the premise that implementation processes benefit from being well-prepared and structured [42]. We have not been able to identify any AI-related studies that have investigated how or to what extent the quality of the implementation process influences the outcomes.

The starting point for the AI-QIF is the QIF, a widely used process model in implementation science. Process models have been developed in this field to facilitate implementation processes [21]. There are many process models, but most share key characteristics with the QIF. To some extent, process models present an ideal view of implementation processes as a structured, linear process that proceeds stepwise in an orderly fashion. Process models aim to create favorable conditions for implementation processes [21]. However, researchers behind many models emphasize that the actual process is not necessarily sequential. The researchers behind the QIF [24] observed that “quality implementation does not always occur in the exact sequence of [the steps].”

The importance of planning ahead of the actual implementation is evident in the QIF; 10 of the 14 steps described in this model are to be carried out before the actual implementation begins. Another common feature of process models is the recognition that context matters; hence, there is a need for adaptation of implementation to local conditions and circumstances [21]. The AI-QIF does not describe specific solutions or pinpoint particular actions. The focus of the framework is on “what” should be done and considered, not on exactly “how” this should be done. Contextual conditions and the characteristics of various AI-based applications are likely to differ considerably, making it difficult to provide a cookbook-style guide.

Following on from repurposing the QIF for use in an AI implementation context (phase I), 2 development phases (phases II and III), and pilot-testing of a prototype (phase IV), we will investigate the barriers and facilitators to using the AI-QIF under routine conditions in health care (phase V). Numerous studies have documented barriers to AI implementation, but we have not identified any studies exploring how AI implementation processes can be supported by using a framework such as the QIF. The use of the QIF can be seen as an implementation strategy intended to facilitate the implementation. Strategies in implementation science are defined as “methods or techniques used to enhance the adoption, implementation and sustainability of a clinical program or practice” [43]. There is wide recognition in implementation science that successful implementation requires efficient strategies to support implementation processes [18].

The development of the AI-QIF will be guided by cocreation principles to create new value together with stakeholders [30] and by incorporating lessons learned from cases of AI implementation in health care practice. The development of the AI-QIF will involve stakeholders such as QIF experts (phase I), health care practitioners and developers (phase II), AI experts (phase III), health care managers (phases II and III), and presumptive health care users of AI-based applications (phases IV and V).

The cocreation process of the project will draw on both research-based and practice-based knowledge. Research-based knowledge is explicit, primarily articulated in different types of text, with the aim of achieving improved understanding or explanation of problems. Practice-based knowledge, on the other hand, tends to be tacit, gained through experience, and expressed through action rather than words, with finding solutions to problems an important aim [44]. Research-based knowledge makes it possible to move beyond the specific “here-and-now” circumstances of a current situation, for example, AI implementation in health care. At the same time, practice-based knowledge is important to account for practical judgment and contextually adapt solutions, for example, the AI-QIF, thus facilitating flexibility and usability. Integrating the 2 forms of knowledge makes it possible to challenge taken-for-granted assumptions, thus broadening the views and enabling unexpected, creative solutions [44].

Some methodological considerations must be accounted for. The framework development is based on data from Sweden, which might negatively affect the generalizability of the results to other countries. Furthermore, much of the development of AI-QIF will take place in the regional setting of Region Halland by researchers from Halmstad University, potentially further restricting the generalizability of the framework. However, national AI and health care developers and experts will be involved in phase III, while phase V is based on the use of AI-QIF in 5 regions in Sweden, thus increasing the generalizability in a Swedish context. In addition, the framework development will be carried out by an experienced multidisciplinary research team, which will strengthen the feasibility of the study and ascertain a comprehensive and robust process. It is also a strength that AI-QIF will be based on the existing and widely used and versatile QIF process model. Cocreation is a guiding principle in the project that will benefit the development since research has shown that cocreation tends to improve the quality, usability, and social acceptance of new solutions [23]. However, there are also challenges with cocreation processes. The literature on the implementation of new technologies in health care suggests that the complexity of technologies can slow down cocreation processes [22,23].

In conclusion, the AI-QIF described in this study protocol aims to facilitate the implementation of AI-based applications in health care based on the premise that implementation processes benefit from being well-prepared and structured. The framework will be coproduced to enhance its relevance, validity, usefulness, and potential value for application in practice.

## Abbreviations

AI: artificial intelligence
AI-QIF: Artificial Intelligence-Quality Implementation Framework
COM-B: Capability-Opportunity-Motivation-Behaviour
QIF: Quality Implementation Framework

## Data Availability

The data sets generated during or analyzed during this study are available from the corresponding author on reasonable request.
